# Wireless‐Powered Electrical Bandage Contact Lens for Facilitating Corneal Wound Healing

**DOI:** 10.1002/advs.202202506

**Published:** 2022-09-08

**Authors:** Qianni Wu, Cheng Yang, Wan Chen, Kexin Chen, Hui‐jiuan Chen, Fanmao Liu, Dong Liu, Haotian Lin, Xi Xie, Weirong Chen

**Affiliations:** ^1^ State Key Laboratory of Ophthalmology Zhongshan Ophthalmic Center Sun Yat‐sen University Guangzhou 510060 China; ^2^ State Key Laboratory of Optoelectronic Materials and Technologies School of Electronics and Information Technology The First Affiliated Hospital of Sun Yat‐sen University Sun Yat‐sen University Guangzhou 510006 China

**Keywords:** bandage contact lens, cornea wound healing, electrotherapy, wireless powered

## Abstract

Corneal injury can lead to severe vision impairment or even blindness. Although numerous methods are developed to accelerate corneal wound healing, most of them are passive treatments that rarely participate in controlling endogenous cell behaviors or are incompatible with nontransparent bandage. In this work, a wireless‐powered electrical bandage contact lens (EBCL) is developed to generate a localized external electric field to accelerate corneal wound healing and vision recovery. The wireless electrical stimulation circuit employed a flower‐shaped layout design that can be compactly integrated on bandage contact lens without blocking the vision. The role of the external electric field in promoting corneal wound healing is examined in vitro, where the responses of directional migration and corneal cells alignment to the electric field are observed. The RNA sequencing (RNA‐seq) analysis indicates that the electrical stimulation can participate in controlling cell division, proliferation, and migration. Furthermore, the wireless EBCL is demonstrated to accelerate the completed recovery of corneal wounds on rabbits’ eyes by electrical stimulation, while the control group exhibits delayed recovery and obvious corneal defects. As a new generation of intelligent device, the wireless and patient‐friendly EBCL can provide a promising therapeutic strategy for ocular diseases.

## Graphical abstract

Schematic of the wireless‐powered electrical bandage contact lens (EBCL) promoting cornea wound healing is discussed here. External electric field produced by the wireless EBCL worn on patient’ eye is capable of regulating the behavior of corneal epithelial cells, thus accelerating corneal wound repair and the recovery of patient's vision.

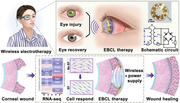

## 1 Introduction

The cornea is an avascular, transparent membrane with a certain curvature located at the outermost layer of the eye, which is at risk for a variety of injuries and diseases.^[^ ^1^, ^2^ ^]^ Corneal injuries account for ≈51% of total serious ocular trauma, and may result in varying degrees of scarring or changes in corneal curvature that can lead to vision impairment.^[^ ^3^ ^]^ Among them, undesired corneal epithelial defects would occur after ocular surgery or in the pathological states. For example, patients often suffer from epithelial defects after corneal transplantation or cataract surgery; they have to wear corneal bandage lens or even perform amniotic membrane transplantation to promote corneal epithelial repair.^[^ ^4^, ^5^ ^]^ Moreover, owing to the improper metabolism in diabetic patients, they are prone to suffer from recurrent corneal epithelial defects,^[^ ^6^ ^]^ which have a poor recovery rate when treated using normal bandage contact lenses (BCLs), resulting in corneal opacification, neovascularization, and subsequent compromised vision. Furthermore, due to the presence of numerous nerves, the corneal epithelial defects are associated with intense pain that causes discomfort and inconvenience to patients.^[^ ^7^, ^8^, ^9^, ^10^ ^]^ Several clinical attempts have been developed to accelerate and improve wound healing, including the use of lubricants, autologous serum, drug‐elution bandage, bandage contact lens, and amniotic membrane transplantation.^[^ ^10^, ^11^, ^12^ ^]^ However, applying gauze and bandage to the injured eye does not allow for direct observation of the corneal wound recovery and intraocular inflammatory reactions; besides, it affects the patient's aesthetic appearance. More importantly, most of these methods are passive treatments that rarely participate in controlling endogenous cell behaviors.^[^ ^13^, ^14^ ^]^ There is an urgent clinical need for a patient‐friendly treatment that can actively and effectively promote corneal repair.

Oriented cell division and migration are essential for wound healing. Exactly, an endogenous electric field, which is produced by ion transporters, channels, and pumps to generate and maintain a transepithelial potential difference, has been proven to participate in controlling cell division and migration.^[^ ^15^, ^16^, ^17^ ^]^ The electric field can promote orderly corneal healing with controlling cell electrical signals. According to the previous studies, the transepithelial potential difference was up to 40 mV, whereas it falls down to 0 mV in wound, thus creating a laterally oriented electric field extending 0.5–1 mm away from the wound.^[^ ^18^, ^19^ ^]^ Herein, taking advantage of electric stimulation for corneal wound healing could imitate the natural wound‐healing mechanism of the endogenous electric field to facilitate epithelial repair. As a noninvasive and active therapeutic method, it is capable of minimizing adverse effects and increasing its efficiency. In the past decades, an external electric field has been used to simulate the endogenous electric field to evaluate their effects on cultured corneal epithelial cells (CECs).^[^ ^20^ ^]^ For instance, Soong et al. found that the externally direct current (DC) electric field could affect the morphology and alignment of corneal epithelial cells in vitro, which is promising in the enhancement of corneal wound healing.^[^ ^21^ ^]^ The electric field at a wound can control the orientation of cell division with the long axis perpendicular to the electric field vector, and regulate the rate of wound healing by changing the electric field strength.^[^ ^22^, ^23^, ^24^, ^25^, ^26^ ^]^ Moreover, Zhao and co‐workers provided a unique approach of pharmacologically enhancing the current to promote corneal wound healing. They used aminophylline and chloride‐free solution to enhance corneal wound current, which is effective in driving cells from the edge of wound to the center.^[^ ^27^ ^]^ Although the influence of the electric field can be significant for corneal wound healing,^[^ ^28^, ^29^, ^30^, ^31^ ^]^ there is still a lack of electrotherapy suitable for clinical application.

Recently, wearable devices, which provide an important and friendly approach for real‐time monitoring of patient's health and in situ medical treatment, have developed rapidly facilitated by the flexible electronics and micro/nanofabrication technologies.^[^ ^32^, ^33^, ^34^ ^]^ Wearable devices, such as smart contact lens and implantable electroceuticals, have made progress in physiological and biochemical signal sensing, as well as in drug delivery for disease treatment.^[^ ^35^, ^36^, ^37^, ^38^, ^39^, ^40^ ^]^ Furthermore, electronic bandages based on electrical and photothermal stimulations or electrical promotion of tissue repair have been applied to achieve long‐term and continuous treatments, as well as intelligent regulation of treatment conditions.^[^ ^41^ ^]^ For example, Lin and co‐workers prepared self‐powered and photothermal electronic skin patches to accelerate wound healing by synergistically utilizing electrical stimulation and photothermal heating.^[^ ^42^ ^]^ In another example, a universal motion‐activated and wearable electrical stimulation device that can effectively promote hair regeneration has also been developed, which provides a non‐pharmacological physical approach for hair loss treatment.^[^ ^43^ ^]^ Moreover, Wang and co‐workers reported an efficient electric bandage based on wearable nanogenerators for facilitating skin wound healing by direct electrical stimulation.^[^ ^44^ ^]^ These studies demonstrated that combining electronic devices with medical bandages can effectively promote wound healing or tissue regeneration. However, currently available electronic therapeutic bandages are primarily based on wire‐powered devices. It is extremely challenging to integrate highly miniaturized wireless electronics for wound recovery in contact lens with small, curved surface and to avoid nonspecific heat effects from the wireless power.

In this work, a wireless‐powered electrical BCL (EBCL) was developed for in situ generation of an external electric field to stimulate the corneal epithelial cells, thus accelerating the repair of corneal wound and the recovery of patient's vision. The flower‐shaped layout design of the wireless electrical stimulation circuit enabled precise and compact integration on limited area of bandage contact lens without vision blockage. Wireless power transfer (WPT) was adopted and optimized to power the contact lens for the formation of an electrical field on the corneal surface, while the thermal safety on eyes of this wireless system was demonstrated. The role of the external electric field in promoting corneal wound healing was examined on human cornea epithelial cells (HCECs) in vitro, where directional migration and alignment of corneal epithelial cells response to the electric field were observed. The differences in gene expression examined by RNA sequencing (RNA‐seq) analysis indicated that electrical stimulation could potentially participate in controlling cell division, proliferation, and migration of HECEs. Furthermore, the wireless EBCL was demonstrated to accelerate the completed recovery of corneal wounds on rabbits’ eyes in vivo after two cycles of treatment with electrical stimulation, while the control group exhibited delayed recovery and obvious corneal defects. The wireless, wearable, and noninvasive features of the EBCL endowed a new opportunity to facilitate the repair of corneal injuries and prevention of secondary ocular disease or vision impairment due to corneal injuries.

## 2 Results and Discussion

Soft bandage contact lens (BCL) conformally interfaced with the cornea is beneficial for protecting corneal damaged epithelium from mechanical erosion caused by eyelid blink, thus providing protective space for corneal re‐epithelialization. BCL was approved for clinical use in the 19th century and is now commonly used to treat a variety of ocular surface diseases, including corneal epithelial defects, corneal erosions, and dry keratitis. Some patients who suffer from persistent corneal epithelial defects or severe dry eye after ocular surgery also require corneal bandage lenses to relieve pain and promote corneal epithelial repair. However, many patients remain unresponsive to bandage lens treatment and still require reoperation for amniotic membrane masking. To solve these problems, we constructed wireless power supply elements on the surface of CBL to stimulate corneal epithelial cell proliferation and migration and promote corneal tissue repair by generating an electric field. Our wireless‐powered EBCL contained the following key advantages: 1) various features of the BCL platform include softness, re‐usability, noninvasiveness, and wireless operations, which are compatible with the clinical applications and the daily life of patients; 2) rational circuit designs and compact structural layout enable precise integration and on‐demand electrical stimulation modulus in limited area of bandage contact lens without vision blockage; 3) the bandage system with electrical interface is desirable for programming and on‐demand stimulation; 4) the fabrication process of EBCL is compatible with the existing circuit board manufacturing process, suggesting the potential for large‐scale and cost‐effective manufacturing.

**Figure 1:**
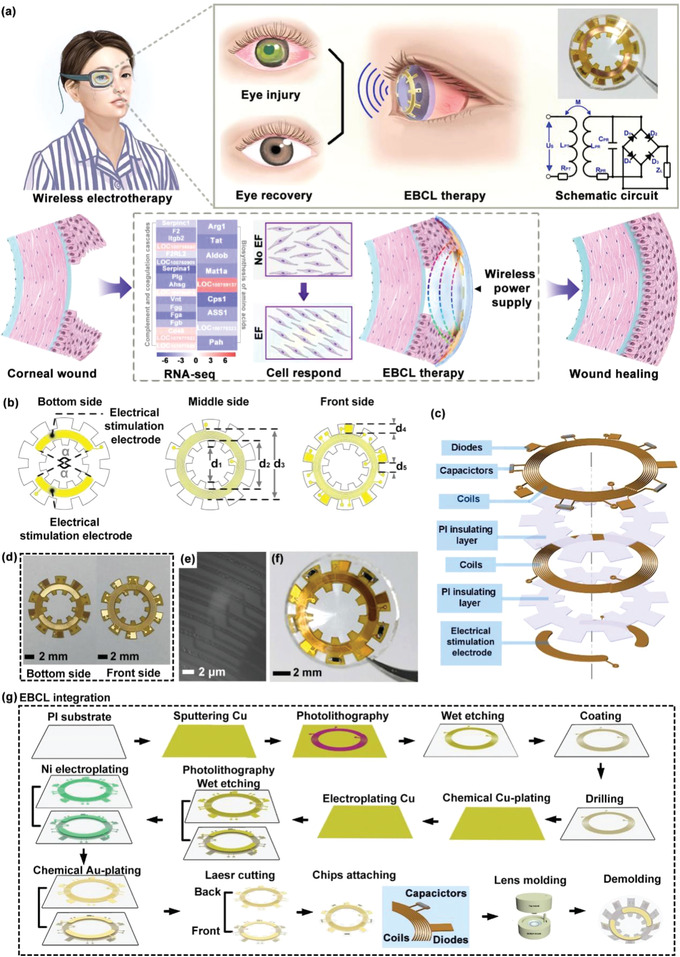
a) Schematic of a significantly accelerated corneal wound recovery under the influence of an external electric field produced by the wireless‐powered EBCL. The external electric field participated in regulating the alignment and migration of corneal epithelial cells, ultimately promoting corneal wound recovery. b–g) Schematic of the EBCL's design and fabrication process: b) layout design, c) structure and d) photograph of the wirelessly electrical stimulation circuit; e) microscopic image of the coils deployed on wirelessly electrical stimulation circuit; f) photograph of the EBCL; and g) schematically illustrated the EBCL fabrication process that employed a printed circuit process coupled with the cast‐molding method. Cu film was grown on a flexible and clean polyimide (PI) substrate by magnetron sputtering, patterned via photolithography, and etched by FeCl_3_ solution. During this process, another layer of PI film was adopted to build multilayer structure, and drilled by laser to fabricate conductive through holes. Moreover, Ni/Au was deposited on Cu electrodes to enhance the biocompatibility of the circuit. Afterward, the sample was shaped by laser according to the layout design, integrated with capacitor and diodes, and integrated with contact lens.

Wireless power transfer is an important energy transmission technology.^[^ ^45^ ^]^ When alternating current (AC) voltage is applied to the transmitting coil in the eyeglass, the current through the transmitting coil will build an oscillating magnetic field. The oscillating magnetic field across the receiving coil is capable of inducing an alternating voltage and current in the receiver circuit integrated in the EBCL.^[^ ^46^ ^]^ In order to regulate the working frequency, a chip capacitor was connected with the receiving coil according to the following equation^[^ ^47^, ^48^ ^]^

(1) $$f={\left(2\pi \sqrt{LC}\right)}^{-1}$$

where *f*, *L*, and *C* represent the frequency, inductance, and capacitance, respectively. The alternating voltage acquired by receiving coil was converted to direct voltage by a bridge rectifier composited of four Schottky barrier diode chips.^[^ ^49^ ^]^ Next, direct voltage could build locally directional electric field on the corneal surface to stimulate corneal epithelial cell. The related parameters of the EBCL's structure are illustrated in Figure S2 (Supporting Information). The wireless‐powered electrical stimulation circuit employed a flower‐shaped layout design (**Figure** **1**b; Table S1, Supporting Information) that enables robust mechanical interlocking between the flexible circuit and the contact lens. The flower‐shaped layout design of the wireless‐powered electrical stimulation circuit enables a rational structure to install multiple electronic components in the rim region of the contact lens. The front side of the circuit (right image of Figure 1d) embedded in the contact lens possessed coils (Figure 1e) connected with the chip capacitor and diodes for wireless power harvesting and rectification, respectively. The electrical stimulation electrodes (left image of Figure 1d) on the bottom side of the flexible circuit were exposed and would be in contact with the cornea for the formation of electrical field to promote the healing of corneal epithelial wound. The flower‐shaped layout design ensured an open vision window larger than the pupil's size in the center without blocking the visions of wearers. As shown in Figure 1g, a printed circuit process coupled with the cast‐molding method was employed for the fabrication process of the wireless‐powered EBCL. For the wirelessly electrical stimulation circuit, Cu film (≈100 µm) was deposited on a flexible polyimide (PI) substrate and patterned via photolithography and wet etching. Then the sample was covered with another PI layer for insulation, which was drilled by laser to form through holes. The Cu film was further deposited to cover the dual surface and holes of the sample. After photolithography and the wet etching process, the Cu coils and stimulation electrodes were patterned on the surface of the flexible PI substrate. Sequentially, surface electrodes were further covered with Ni/Au to enhance the biocompatibility of the circuit. Afterward, the PI substrate was shaped by laser according to layout design. Finally, the flexible circuit was soldered with capacitor and diode chips, embedded into the polydimethylsiloxane (PDMS) lens via cast‐molding technique. This was followed by integration of a small‐sized multilayer ceramic chip capacitor (length = 1.05 mm, width = 0.45 mm, and thickness = 0.55 mm) on the flexible circuit. The tiny electrical components with considerable capacitive reactance deployed on the circuit have the ability to counteract the inductive reactance of the coils at 950 kHz. This configuration is expected to significantly reduce the impedance of the circuit, which can minimize power consumption and improve power utilization. Moreover, four Schottky barrier diode chips (length = 1.00 mm, width = 0.60 mm, and thickness = 0.55 mm) were integrated on the flexible circuit to form a bridge rectifier (Figure S3, Supporting Information) by a low‐temperature lead‐free solder at a temperature of 220 °C. This rectifier could convert alternating voltage to direct voltage. Finally, the prepared flexible circuit was embedded into the PDMS lens via cast‐molding technique, illustrated in Figure S4 (Supporting Information). The fabrication process of the wireless‐powered EBCL was compatible with that of the standard printed circuit board process, suggesting the feasibility of large‐scale manufactures of this wearable biomedical device. An antenna system of WPT transmitters was fabricated, which could transfer power to the WPT receiver of the EBCL.

**Figure 2:**
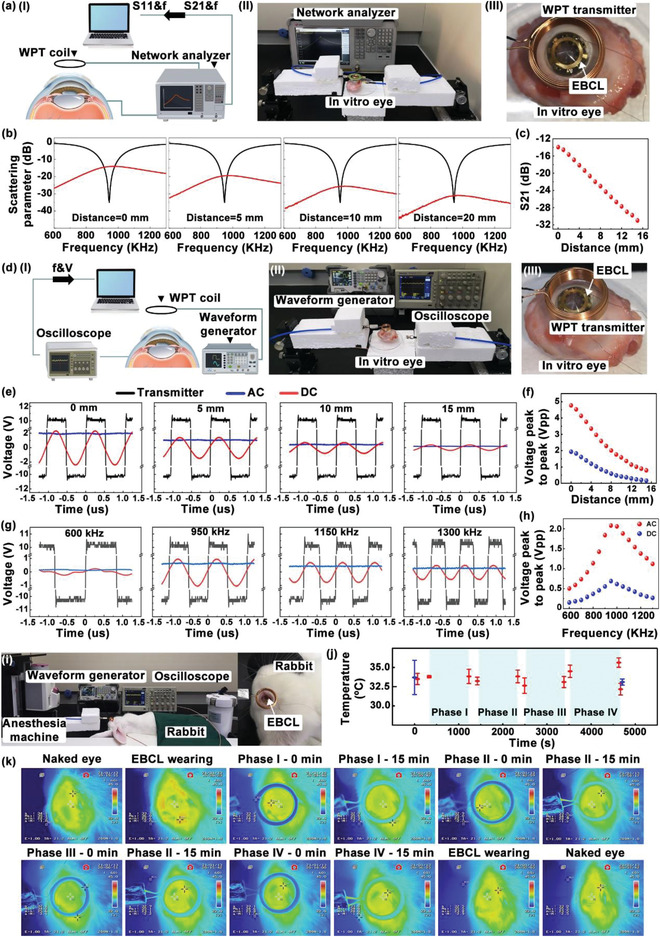
Characterization of the WPT and rectification circuit. a‐I) Schematic diagram and a‐II) optical image of the scattering parameters measurement of the WPT system. a‐III) Photograph of the WPT system. The contact lens integrated with the WPT receiver was worn on in vitro porcine eye. b) Scattering parameters’ measurement of the WPT system. Return loss (S11) recorded from the WPT transmitter was plotted as black curves. Insert loss (S21) recorded from the WPT receiver was plotted as red curves. c) The influence of radiation distance and frequency on wireless voltage transfer. d‐I) Schematic diagram and d‐II) photograph of the scattering parameters’ measurement of the WPT system. d‐III) The contact lens integrated with the WPT receiver and aligned over transmitter along the identical axis was worn on in vitro porcine eye. e) Voltage waveform collected on the coils (red curves) and electrical stimulation electrodes (blue curves) of the WPT receive circuit under different radiation distances with 20 Vpp square voltage applied on the transmitter (black curves). f) Peak‐to‐peak voltage before and after rectification was plotted as a function of distance. g) Voltage waveform collected on the coils and electrical stimulation electrodes of the WPT receive circuit under different radiation frequencies with 20 Vpp square voltage applied on the transmitter. h) Peak‐to‐peak voltage before and after rectification was plotted as a function of frequency. Thermal characterization during animal experimentation. i) Left: photograph of wireless‐powered electrical stimulation during animal experimentation. Right: Wireless‐powered bandage contact lens worn on cornea of the anesthetic rabbit. j) Statistical temperature of the bandage contact lens and cornea during animal experimentation. k) Infrared images of rabbit eye and bandage contact lens during animal experimentation.

In this work, WPT technology was adopted to power the contact lens for the formation of an electrical field on the corneal surface. WPT is a promising electrical energy transmission strategy, which could improve the convenience of wearable electronics, especially for the bandage contact lens device. To evaluate the power transfer performance, the optimal operation frequency and the distance separation between the EBCL and WPT transmitter were examined. Before tests, the WPT transmitter and WPT receive circuit integrated in contact lens were connected to different ports of the network analyzer (**Figure** **2**a‐I,a‐II). Moreover, the WPT transmitter was aligned over the contact lens with an identical axis (Figure 2a‐III). The scattering parameters of the wireless system were recorded under different radiation distances ranging from 0 to 15 mm with a step of 1 mm (Figure 2b; Figure S5, Supporting Information). Results indicated that the WPT transmitter and the receive circuit could be coupled well in the frequency of 950 kHz. The insert loss (S21) of the WPT receive circuit on 950 kHz was collected and plotted as the function of radiation distance (Figure 2c). It demonstrated that S21 decreased linearly with the increased radiation distance, implying that the wireless coupling between the transmitter and receiver weakened with the increase in radiation distance. In order to explore the influence of radiation distance on wireless voltage transmission, the WPT transmitter connected to a waveform generator was aligned over the WTCL with identical axis (Figure 2d‐I,d‐II), while the WPT receivers were connected to an oscilloscope. To build stable magnetic coupling between the transmitter and receiver, a square voltage wave of 20 Vpp was produced from the waveform generator to the WPT transmitter.^[^ ^50^ ^]^ Correspondingly, the voltage on the coils and electrical stimulation electrodes of WPT receive circuit were recorded (Figure 2e; Figure S6, Supporting Information). Voltage waveform demonstrated that the voltage collected on electrical stimulation electrodes of the WPT receive circuit showed direct voltage, implying that the induced voltage on the receiver was rectified, which could form stable electrical field between the stimulation electrodes and improve cell‐directed migration. The direct voltage was significantly lower than that of the established WPT system that was developed for the power glucose sensor and light‐emitting diodes.^[^ ^36^ ^]^ However, the moderate direct voltage is capable of avoiding biological tissue damages caused by the electrochemical burns and electroporation effect.^[^ ^51^, ^52^ ^]^ Then the voltage on the coils and electrical stimulation electrodes of the WPT receive circuit at different radiation distances were recorded (Figure 2f). This figure demonstrated that the wireless transmitted voltage decreased gradually with the increased radiation distance, which matched with the result mentioned in Figure 2c. To verify the optimal operation frequency of the WPT system, 20 Vpp of square voltage wave with different frequencies (from 600 to 1300 kHz with a step of 50 kHz) was exerted on the WPT transmitter. The contact lens integrated with the WPT receiver worn in vitro on a pig's eyeball was aligned over the WPT transmitter with identical axis. A distance of 6 mm was set between the WPT transmitter and receiver. The voltage on the coils and electrical stimulation electrodes of the WPT receive circuit are shown in Figure 2e and Figure S7 (Supporting Information). The results demonstrated that the nonsinusoidal voltage waveform was collected on the receiver coils when 20 Vpp of square voltage wave with lower frequency (600 kHz) was exerted on the transmitter. It means that the square voltage with a frequency of 600 kHz could not build reliable coupling with the receiver. With the increase in square voltage frequency, the voltage collected on the receiver coils exhibited sinusoidal voltage waveform. Besides, voltage recorded on the electrical stimulation electrodes of the WPT receive circuit exhibited direct voltage features, revealing that the rectification circuit module possessed the abilities to convert alternating voltage into direct signal. This could potentially broaden the application in biomedical stimulation if direct current was required. After that, the voltage on the coils and electrical stimulation electrodes of the WPT receive circuit at different operation frequencies are recorded in Figure 2h. This result exhibited that the wireless transmitted voltage enhanced gradually with the increased operation frequency and reached the peak at 950 kHz. Then the values of voltage decreased as the operation frequency increased further. It revealed that 950 kHz represented the optimal operation frequency of the WPT system, which matched with the result mentioned in Figure 2b. The wireless electrical stimulation is likely to generate undesirable thermal effects on the eyes. The ocular temperature fluctuations that occurred during wireless‐powered electrical stimulation were recorded using an infrared camera, as shown in Figure 2i–k and Figure S8 (Supporting Information). Figure 2k demonstrates infrared images of ocular surface of anaesthetized rabbit's eye during wireless operation. Statistical analysis showed that the temperatures of the corneal surface and lens at the initial stage were 33.10 ± 1.11 and 33.00 ± 0.36 °C, respectively. The maximum temperature was 33.50 ± 0.4 °C, which demonstrated the thermal safety of this wireless system that possessed negligible thermal changes in the rabbit's eye during operation.

**Figure 3:**
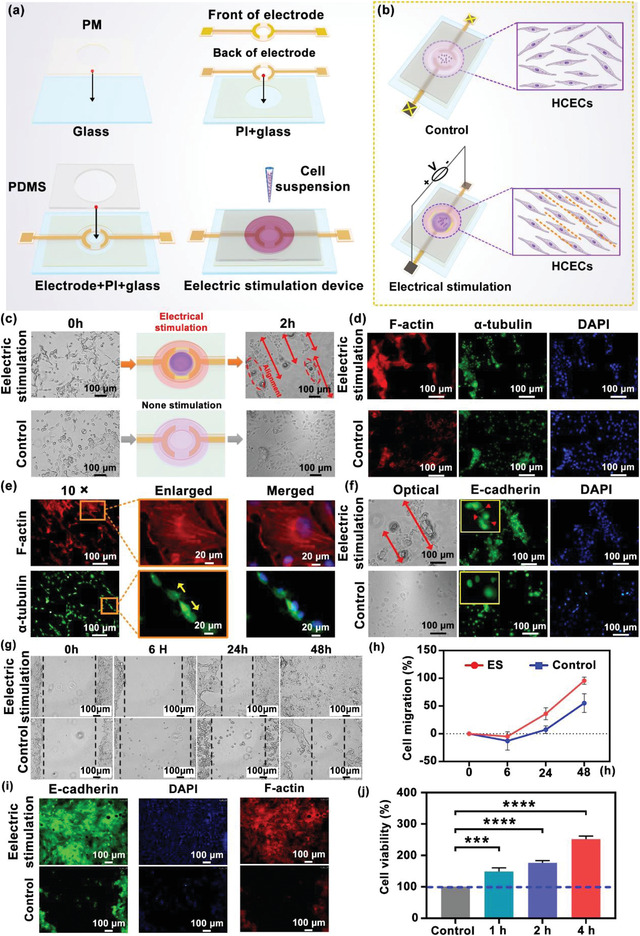
Influence of the applied electric field on cultured human cornea epithelial cells (HCECs). a) Fabrication of the electric stimulation chamber. b) Schematic illustration of HCECs cultured in the electric stimulation chamber connected and disconnected to DC output voltage. c) Morphology of the cultured HCECs at different time points (0 and 2 h) with and without (control) electrical stimulation. Obvious morphological and directional responses of the HCECs were observed in the electrical stimulation treated group (parallel to the red double arrow line) at 2 h. d) Fluorescent images of the HCECs labeled with F‐actin (red) and *α*‐tubulin (green). Scale bars = 100 µm. e) Enlarged fluorescent images of the HCECs labeled with F‐actin (red) and *α*‐tubulin (green); the yellow arrows point to the direction of cell division. Scale bars = 20 µm. f) Optical images and immunofluorescence staining for E‐cadherin, the red double arrow lines indicate linear alignment and directional migration of the HCECs, and the red arrows point to the junction of the HCECs. Scale bars = 100 µm. g) Representative images of the gap at 0, 6, 24, and 48 h in the scratch assay. h) Percent cell migration at different time points (0, 6, 24, and 48 h). i) Immunofluorescent staining of E‐cadherin in the electric stimulation and control group at 48 h. Scale bars = 100 µm. j) Cell viability at different times of electric stimulation (1, 2, and 4 h). ***p < 0.0005, **** *p* < 0.0001.

In order to investigate the role of an external electric field in promoting corneal wound healing, electrical stimulation was performed on cultured HCECs in vitro. To facilitate experimental operation, here the electrical stimulation was applied by connecting stimulation electrodes to the power supply rather than in a wireless manner. Moreover, the electrical stimulation circuit was flattened on a transparent glass substrate for cell culture (**Figure** **3**a,b; Figure S9, Supporting Information), rather than on a curved contact lens. A PDMS well was attached on top of the stimulation electrodes as the container of the cell culture medium. Cell suspensions were supplemented into the culture well, and the cells would adhere to the substrate and grew as a single layer. The orientation of the HCECs would be random in untreated culture conditions. When an electric field was applied, the pair of stimulation electrodes would generate the electric field in parallel to the cell culture layer. This cellular electrical stimulation device can mimic the essential physical features of the in vivo states of EBCL worn on the cornea, and present a more convenient experimental setup for studying the cell response to the electric field in vitro. The effect of electrical stimulation on the HCECs was studied by observing the cell alignment and migration under the electric field in the cellular electrical stimulation device. After the 24 h incubation of HCECs, the stimulation electrodes were connected to a waveform generator to produce a DC electric field of 1 V cm^−1^ for 2 h, while the control group was treated without electric stimulation. As shown in Figure 3c, initially, the HCECs aligned randomly. After the electrical stimulation treatment, the alignment of a certain area of cells that localized in the area close to the electrodes exhibited a polarized profile after 2 h long culturing, approximately parallel to each another along the indicated red arrow line. In contrast, the morphology and alignment of HCECs in the control group barely showed any change within 2 h.

To further investigate the migration and proliferation of HCECs, the rhodamine‐conjugated phalloidin was utilized to stain the cellular morphology and alignment by labeling the filamentous actin (F‐actin) of the cells with red fluorescence; furthermore, the immunofluorescence staining was employed to show the nuclear division via labeling *α*‐tubulin with green fluorescence. As shown in Figure 3d, in the electrical stimulation‐treated group, the expression of F‐actin was significantly increased. Meanwhile, the HECEs treated with electrical stimulation display certain degrees of alignment profile along the indicated red arrow line, which was nearly perpendicular to the electric field vector. Moreover, a thickened F‐actin bundle parallel to the long axis was observed in the cells treated with electrical stimulation (Figure 3e). Importantly, actin polymerization aligned perpendicularly to the electric field vector has been reported to be the key driving force for the electric field guided cell migration.^[^ ^19^, ^23^ ^]^ The alignment of cells was mainly caused by the electric‐field‐induced asymmetric polymerization of the F‐actin polymerization, which maintained the cell morphology. On the other hand, the immunofluorescence staining results exhibited that the direction of cell division was perpendicular to the electric field vector. In addition, compared with the control group, the expression of intercellular connexin E‐cadherin was increased in the electrical stimulation‐treated group (Figure 3f). These results indicated that the applied electric field would induce the alignment and migration of HCECs without manipulating the cells mechanically, which is an important indication of the feasibility of facilitating the restoring of the epithelial barrier and promoting wound healing by the electric field. The directional migration and alignment of the corneal epithelial cells’ response to an electric field are consistent with that of several studies that have been reported. For example, Zhao et al. also found that corneal epithelial cells rearrange directionally in response to an electric field with the long axis of the cells being perpendicular to the electric field vector. However, the mechanism of electrical stimulation on CECs is not clear and may be related to asymmetric activation of cell surface receptors through cell signaling pathways.^[^ ^15^, ^20^, ^25^ ^]^ Since HCECs displayed obvious cytoskeletal changes after treatment with electrical stimulation, we further test the influence of electrical stimulation on cell migration by a scratch assay. The HCECs were cultured in the electrical stimulation device to form a monolayer, then the scratch wounds were made in the center by a cell scratch scraper. An optical microscope was utilized to observe and photograph the scratch wounds. The experimental group received electrical stimulation for 2 h (5 min interval followed by 15 min stimulation), while the control group received no treatment. The cell recovery in the scratch area was dynamically monitored on 0, 6, 24, and 48 h, which was further calculated to analyze to healing rate. As shown in Figure 3g, the wound healing rate in the electric‐field‐treated HCEC culture was much faster and more effective than that in the control group of the HCECs. After 48 h of scratch formation, the‐electric field‐treated HECEs between the two regions of scratch migrated to cover the scratch area. The wound area in the electric‐field‐treated HECE culture was observed to be completely recovered (≈100%) after the 48 h long culture. However, the wound area of the control group without electric field treatment was slowly reduced to ≈41.2%, which was not completely recovered even after the 48 h long culture (Figure 3h). Through the labeled staining of connexin and skeletal protein, we found that under the influence of the electric field, a confluent monolayer with tight intercellular adhesion was formed after the closure of the wounds (Figure 3i). Besides, the stretched actin filaments perpendicular to the electric field vector were also observed, suggesting the directional arrangement of HECEs. The effect of electric field on promoting cell proliferation was evaluated using the CCK‐8 assay by comparing the cell viabilities to control group (Figure 3j). A DC electric field of 1 V cm^−1^ was applied on the HECEs for electrical stimulation for 4 h. The result showed that the cell viability was significantly improved in the electric‐field‐treated group for 1, 2 and 4 h, and that of electric field‐treated group at these three time points was quantified to be 148.33% ± 11.97%, 176.33% ± 7.3%, and 251.57% ± 9.94% compared to the control group, respectively. The cell viability of electric‐field‐treated group was ≈2.51 times higher than that of the control group after 4 h culture. The higher cell viabilities revealed that the applied electric field could promote the proliferation of the HECEs. Furthermore, the corneal epithelial wound healing was found to be closely related to the guidance of endogenous electric fields. Consistent with the previous study, our experimental results also demonstrated that electric fields could guide directed cell migration in a monolayer cell model of CEC wound repair, but the exact mechanism has not been clarified.^[^ ^53^ ^]^

**Figure 4:**
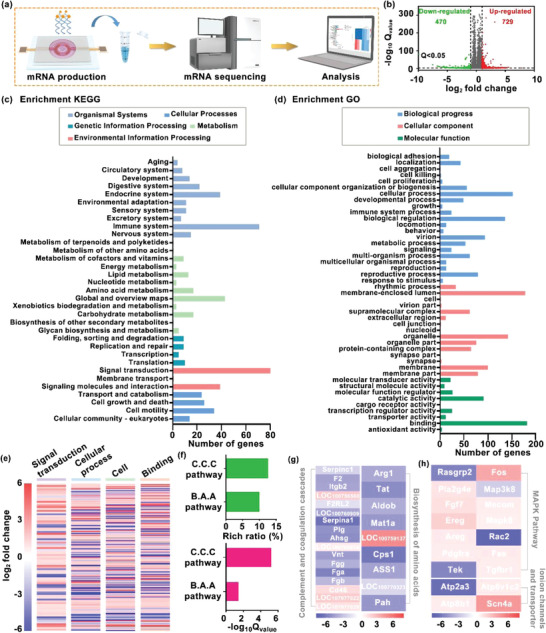
Study of mechanism of epithelial wound healing treated by electrical stimulation. a) Schematic diagram of the mRNA sequencing procedure. The experimental group was given electrical stimulation treatment, while the control group was not given any interventions and then transported to the company at a low temperature for mRNA detection. b) Volcano plots displayed 729 significantly upregulated genes and 470 significantly downregulated genes whose *Q* value is <0.05 and |log _2_| fold change ≥ 1 compared the electrical stimulation group with the control group. c,d) The number of differentially expressed genes (DEGs) analyzed by KEGG and GO. The KEGG analysis showed that the changes in DEGs were mainly involved in the 33 classifications related to biochemical metabolic and signal transductions, and there were 45 gene classifications that changed linked to the cellular process by GO analysis. e) Major changes of gene expression induced by electrical stimulation. f) Rich ratio (%) and *Q*_value_ of genes in the enriched KEGG which are highly related to complement and coagulation cascades (C.C.C.) and biosynthesis of amino acids (B.A.A.) pathways. g,h) Heat maps of the genes encoding complement and coagulation cascades, biosynthesis of amino acids, MAPK pathways, ion channels, and transporter.

Furthermore, to reveal that the genes express differences induced by the electrical stimulation treatment, RNA‐seq analysis was employed (**Figure** **4**a). The experimental group received electrical stimulation for 2 h (5 min interval followed by 15 min stimulation), while the control group received no treatment. After electrical treatments on cells, the messenger RNA (mRNA) inside the cells was extracted from the CECs in each group with TRIzol solution for mRNA detection. Differential gene analysis was performed by the DEGSeq method, and genes with a *Q* value of <0.05 and ǀ log_2_ ǀ fold change≥ 1 were screened as significant differential expressed genes (DEGs). Based on the DEGs investigated in the analysis, Figure 4b presents the volcanic maps of each gene that is upregulated or downregulated by electrical stimulation treatment compared to the control group. The test found that under the standard of *Q*_value_ < 0.05, the number of differential genes of electrical stimulation was 729 significantly upregulated genes, and 470 significantly downregulated genes, indicating that the electric field possessed a statistically significant influence on the HCECs. Additionally, the expression of the contributing genes that responded to the electrical stimulation was further analyzed by kyoto encyclopedia of genes and genomes (KEGG) and gene ontology (GO). The KEGG analysis showed that the changes on the DEGs were mainly involved in 33 classifications related to biochemical metabolic and signal transduction (Figure 4c), and there were 45 gene classifications that changed linked to a cellular process by GO analysis (Figure 4d). Among them, the signal transduction, cellular progress, and cell binding are displayed in Figure 4e, which were the most gene changes in the KEGG and GO analyses. Moreover, the KEGG enrichment analysis showed that DEGs were significantly focused on complement, coagulation cascades (C.C.C.) and biosynthesis of amino acids (B.A.A.) pathway (Figure 4f), whose enrichment rates exceed 10% with *Q*_value_ ˂ 0.05 and the heat maps are displayed in Figure 4g. Besides, the significant gene expression changes of Rasgrp2, Fos, Rac2, Tek, and other 14 genes were related to mitogen‐activated protein kinase (MAPK) pathway (*Q*_value_ ˂ 0.05), which regulated the physiological processes of the cell, including proliferation, differentiation, and migration. The applied electric field can also affect the genes associated with the ion channels and transporter‐linked genes, such as Atp2a3 that is involved in regulating Ca^2+^ transporting, Atp6v1c2 encodes the hydrogen ion transporting, Atp8b1 regulates phospholipid transport, and Scn4a regulates Na^2+^ channel. These results indicated that the electrical stimulation treatment could potentially participate in controlling cell division, proliferation, and migration of the HECEs (Figure 4h).

**Figure 5:**
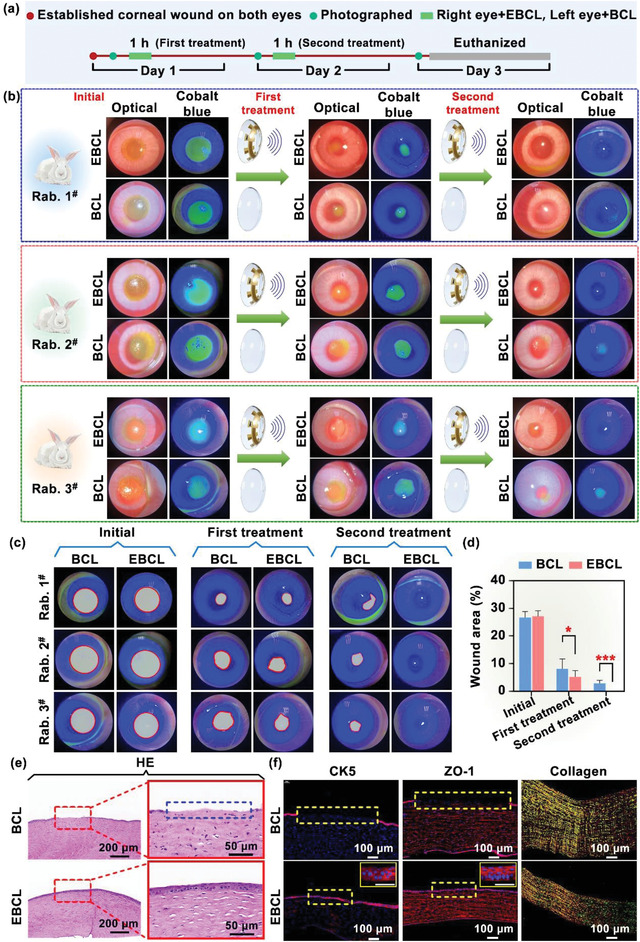
Corneal wound healing under stimulation of the external electric field based on integrated EBCL. a) Procedure of animal experiments, mainly including animal corneal wound modeling, slit lamp microscopic photography, and electrical stimulation treatment. b) Slit lamp microscopic images of a 3 day healing progress for corneal epithelial wounds with the EBCL (OculusDexter, OD) and the pristine bandage contact lens (OclulusSinister, OS). *n* = 3. c) The corneal wound area in both eyes was outlined and calculated by ImageJ software. The result showed that EBCL could significantly facilitate corneal wound recovery. d) Corneal wound area (%) as a function of time with electrical stimulation by the EBCL (red) and the pristine bandage contact lens (blue). *n* = 3. e) Representative of H&E‐stained sections of corneal wound after 3 days with or without electrical stimulation treatment. The blue dashed line shows the area of corneal epithelial defect. f) Immunofluorescent staining of CK5 (corneal epithelial cell marker) and ZO‐1 (intercellular linker protein) presented the entire thickness of the epithelium and intercellular junction, indicating the unclosed wound in the control group (indicated by the yellow dashed box). Scale bars = 50 µm.

The wireless‐powered EBCL was used as a wearable electric field generator to promote corneal epithelial wound healing in rabbit models. First, we established a corneal epithelial wound model on the eyes of New Zealand rabbits (*n* = 3). A corneal epithelial defect model was constructed for both eyes of each rabbit, and the procedure was briefly described as follows: the extent (5 mm diameter circle) and depth of the wound (penetrating the entire epithelial layer to reach the surface of the stroma) were determined by corneal ring drilling, and the epithelial cells were gently scraped away using a lamellar knife. To ensure that the depth and size of the wound are consistent in each eye, the animal eye surgery models were established by the same ophthalmologist. The EBCL with a wireless power transfer receiver was aligned over the transmitter along the identifying axis and was worn on the anesthetized rabbit's right eye for electrical stimulation for 60 min per day (with 1 min interval after every 15 min of stimulation). The waveform generator provided wireless power to generate 950 kHz alternative voltage with 20 V_PP_ of square voltage. In addition, the WPT receivers were connected to an oscilloscope to trace the voltage produced by a wireless contact lens. Meanwhile, the left eye of each rabbit wore on pristine BCL was treated as the control group. Each eye was photographed using a slit lamp after treatment on days 1, 2, and 3, and the wounds were examined and compared (**Figure** **5**a). The rabbit's eyes were stained with sodium fluorescein solution prior to the daily electrical stimulation treatment. After rinsing with sterile saline to remove excess stain, the eyes were observed and photographed under a slit lamp microscope by cobalt blue light. Cell membrane breaks and early apoptotic cells will exhibit yellow–green fluorescence, and normal intact cells will remain unstained, allowing assessment of the extent of corneal epithelial wound.^[^ ^54^ ^]^ The time‐varying healing process of corneal wounds is shown in Figure 5b, where both the optical and cobalt blue images of the cornea during the 3 day healing process were presented. Initially, the wound area accounted for the overall area of cornea was ≈27% after the formation of the corneal wound model on day 1. On the second day, the area of corneal wound of the EBCL group was observed to be reduced to 5.19% ± 2.29%, which is 1.68 folds lower than the control group. After wearing the wireless‐powered EBCL for 3 days with electrical stimulation treatment, the corneal wound was observed to be recovered completely, and the cornea was transparent without edema. In contrast, an epithelial wound with a diameter of 2.24 ± 0.87 mm remained in the corneal center of the control group, which was also accompanied by corneal edema (Figure 5c,d). According to our calculation, the healing rate of corneal wound in the control group was 58 ± 18 µm h^−1^, while the electric stimulation increased the healing rate by 1.79‐fold. This result indicated that the healing rate of corneal wound in the EBCL group increased significantly compared to that in the control group. Besides, Zhao and co‐workers used aminophylline and chloride‐free solution to enhance corneal wound currents and found that the corneal wound healing rate increased by 28% after local administration of aminophylline.^[^ ^27^ ^]^ To the best of our knowledge, it is the first example to integrate electrical stimulation technology with contact lens aiming to ocular wound repair. The current studies mainly focus on skin tissue repair. For example, a programmed and skin‐temperature‐activated electromechanical dressing was prepared for promoting wound healing. This flexible dressing achieves effective wound healing in as short as 4 and 8 days for linear and circular wounds, respectively; moreover, the wound healing rate increased more than 50% compared with the blank control group.^[^ ^42^ ^]^ Moreover, the effects of AC and DC on wound recovery are often related to the specific cells and animal tissues.^[^ ^41^, ^42^, ^43^, ^44^, ^54^ ^]^ In this study, the use of DC was optimized according to the results of cell and rabbit model experiments. Furthermore, the electrical stimulation signal for wound recovery can be regulated according to different cells or tissues.

Afterward, to observe corneal tissue morphology and potential defects associated with the wound, the experimental rabbits were euthanized and the corneas were collected for hematoxylin‐eosin (H&E) staining. As shown in Figure 5e, disorder in the arrangement of corneal stromal cells and corneal edema (indicated as the blue dotted lines) was observed in the control group, demonstrating the existence of the corneal epithelium defect. In contrast, the corneal epithelium layer remained intact and the stromal cells were arranged regularly in the EBCL‐treated eyes. The expression of the cell tight junctional proteins, such as zonula occludens‐1 (ZO‐1), will decrease in pathological conditions.^[^ ^55^ ^]^ To evaluate the remodeling in corneal epithelial cells and the promoted anchoring of tight junction after EBCL treatment, the extent of cell recovery and the tightness of the intercellular junctions were determined by immunofluorescent staining of the corneal epithelial cell marker cytokeratin 5 (CK5) and ZO‐1. According to the immunofluorescence staining results, the wounds were still open in the control group without the expression of CK5 and ZO‐1 after 3 days’ treatment. In contrast, the corneal epithelial layers remained intact and complete with the expression of CK5 in the EBCL group. Furthermore, strong expression of ZO‐1 in the EBCL‐treated group was observed, indicating that the resistance barrier of corneal epithelial was significantly regenerated.^[^ ^56^, ^57^, ^58^ ^]^ Although obvious corneal stromal edema appeared in the control group, no significant difference in the composition of corneal collagen matrix between the two groups was observed (Figure 5f). In summary, the eyes treated through electrical stimulation exhibited a completely healed wound with normal appearing epithelialization, while the control eyes still displayed an obvious epithelial defect. These results demonstrated that the utilization of an external electric field on EBCL could regulate the orientation and frequency of the cell division and proliferation, promote anchoring of cell tight junction, and accelerate the rate of wound healing in vivo. This contact‐lens‐based wireless wearable electric bandage could provide a safe, convenient, and efficient treatment for the corneal wound.

## 3 Conclusion

In conclusion, the concept for the function of electrical stimulation based on a wireless‐powered EBCL was demonstrated to accelerate corneal wound healing and recovery the patient's vision. The EBCL without vision blockage was powered by WPT to generate an electrical field on the corneal surface, thus regulating the directional migration and alignment of the HCECs. Moreover, the EBCL with electrical interface is desirable for programming and on‐demand stimulation. The migration and proliferation of the HCECs were demonstrated to be perpendicular to the electric field vector. Furthermore, the animal experiments demonstrated that the wireless EBCL accelerated the completed recovery of the corneal wounds on the rabbits’ eyes after two cycles of electrical stimulation, while the control group exhibited delayed recovery and obvious corneal defects. These therapeutic effects were attributed to the applied electric field, which participates in controlling the cell division, proliferation, and migration. The wireless, wearable and transparent device provides a highly efficient electrotherapy strategy for ocular disease treatment. Moreover, flexible electronic components with similar mechanical properties will prompt the practical applications of this wearable device, which is the main research direction in wearable semiconductor devices.

## Conflict of Interest

The authors declare no conflict of interest.

## Author Contributions

Q.W. and C.Y. contributed equally to the work. W.‐r.C. and X.X. conceived and designed the experiments. C.Y. designed, fabricated, and characterized the wirelessly electrical stimulation circuit and EBCL. Q.W. and C.Y. performed most of the experiments and analyzed the data. D.L., W.C., K. C., W.C., assisted in some experiments. H.‐j.C., F.L., and H.L. provided technical assistance and guided the experimental design. W.‐r.C. and X.X. supervised the research. Q.W., C.Y., X.X., and D.L. wrote and revised the paper.

## Supporting information

Supporting Information

## Data Availability

The data that support the findings of this study are available from the corresponding author upon reasonable request.
