# Supplementary material for: Wireless‐Powered Electrical Bandage Contact Lens for Facilitating Corneal Wound Healing
Source: Adv Sci (Weinh). 2022 Sep 8;9(31):2202506. doi: 10.1002/advs.202202506 (PMC9631068; doi:10.1002/advs.202202506)
Supplement: Supplementary file 1 — Supporting Information [file ADVS-9-2202506-s001.pdf]

Supporting Information

Wireless-powered electrical bandage contact lens for facilitating corneal wound healing

Qianni Wu[1+], Cheng Yang[2+], Wan Chen[1], Kexin Chen[1], Hui-jiuan Chen[2], Fanmao Liu[2], Dong Liu[1], Haotian Lin[1], Xi Xie[1,2*], Weirong Chen[1*]

[1] State Key Laboratory of Ophthalmology, Zhongshan Ophthalmic Center, Sun Yat-sen University, Guangzhou 510060, China.

[2]State Key Laboratory of Optoelectronic Materials and Technologies, School of Electronics and Information Technology; The First Affiliated Hospital of Sun Yat-sen University, Sun Yat-sen University, Guangzhou, China

*To whom correspondence may be addressed. Corresponding to: Weirong Chen, chenwr_q@aliyun.com; Xi Xie, xiexi27@mail.sysu.edu.cn

[+]These authors contributed equally to the work.

Method

Fabricaiton of wirelessly electrical stimulation circuit.

The fabrication process of the wireless flexible circuit employed a printed circuit process. For the wirelessly electrical stimulation circuit, Cu film with thickness of 100 nm was grown on a flexible and clean polyimide (PI) substrate by magnetron sputtering, and patterned via photolithography, and etched by $FeCl_3$ solution. Then the flexible sample was covered with another layer of PI film, and drilled by laser to form through-holes. After that, Cu film was covered on the dual-surface and holes of the sample by chemical and electroplating. Then Cu coils and stimulation electrodes were patterned on the surface of the flexible sample by photolithography and wet etching process. Sequentially, these surface electrodes were further covered with Ni/Au to enhance the biocompatibility of the circuit. Afterwards, PI substrate was shaped by laser according to layout design. Then, a multilayer ceramic chip capacitor with small size (length = 1.05 mm, width = 0.45 mm, thickness = 0.55 mm) and four Schottky barrier diode chips (length = 1.00 mm, width = 0.60 mm, thickness = 0.55 mm) were integrated on flexible circuit by low temperature lead-free solder at temperature of 220 ℃.

Fabricaiton of EBCL.

Wirelessly electrical stimulation circuit was positioned into the metal mold. Afterwards, transparent liquids (Polydimethylsiloxane (PDMS, Sylgard 184, Dow Corning) and curing agent mixed according to the ration of 10:1) was mixed sufficiently by stirring and degassed by high-speed centrifugation. Then the transparent liquids were immitted into bottom mold. Sequentially, and the bottom mold contained wirelessly electrical stimulation circuit and liquid PDMS was placed in vacuum with pressure of 10 Pa for 30 min to remove residual bubbles. After vacuum treatment (10 Pa, 30 min), the mold was assembled and baked in oven with temperature of 80 °C for 1.5 h. Finally, the top and bottom mold were separated. And contact lens integrated with circuit was detached from mold carefully.

Microscopic image of wirelessly electrical stimulation circuit.

The wireless electrical stimulation circuits were characterized by camera of inverted fluorescence microscope (MF52-N, Guangzhou Micro-shot Technology Co., Ltd, China)

Scattering parameters measurement of the EBCL.

During the measurements, wireless power transmitter coil was immobilized above EBCL worn on porcine eye. The ex vivo organ was posited on cystosepiment board held by a multi-axis stages to adjust the distance between EBCL and wireless power transmitter coil. During the collection of scattering parameter, two ports of vector network analyzer (E5063A, Keysight Technologies, USA) was connected to the wireless power transmitter and coils of EBCL.

WPT performance characterization of the WTCL system.

During the measurements of wireless voltage transfer performance, waveform generator (DG1022, Beijing RIGOL Technology Co., Ltd., China) was adopted as power source for operating the wireless power transmitter. While oscilloscope (TDS2014C, Tektronix, USA) was connected to coils or stimulation electrodes of wireless power receiver circuit in EBCL for recording the AC voltage signal collected wirelessly and the DC voltage signal rectified.

Thermal characterization.

New Zealand white rabbit (2 kg), anesthetized with pentobarbital sodium solution (0.8 ml/kg body weight) through ear venous injections, was covered with blanket to maintain body temperature. Then anesthesia machine was exploited to supply mixture gas (isoflurane and oxygen) via facemask for the rabbit, which could prolong the anesthesia time. After that, EBCL was worn on rabbit's eye. And wireless power transmitter connected to waveform generator was posited above EBCL with the

distance of 6 mm. Square voltage with 20 Vpp at 950 kHz produced from waveform generator was exerted on wireless power transmitter. Infrared camera (T650sc, FLIR Systems, Wilsonille, OR, USA) was exploited to monitor thermal changes of ocular surface tissue, EBCL, and wireless power transmitter during the experimental process.

Human primary corneal epithelial cells culture

Human primary corneal epithelial cells were purchased from ATCC (PCS-700-010). After reviving the frozen cells, the cells were cultured and passaged according to the guidelines of the instructions on the ATCC website..

Fabrication of cellular electrical stimulation device

Fabrication of cellular electrical stimulation device was carried with two sub-steps: electrical stimulation circuit fabrication and device’s assembling. First,

For the electrical stimulation circuit fabrication, Cu film with thickness of 100 nm was deposited on a clean polyimide (PI) substrate by magnetron sputtering. In order to acquire acceptable thickness, electrical plating process was adopted to elevate the thickness of Cu film. Then the Cu film was patterned via photolithography, and etched by $FeCl_3$ solution. After that, Cu film was covered with Ni/Au to enhance the biocompatibility of the circuit. Afterwards, PI substrate was shaped by laser to form cellular electrical stimulation circuit.

For the cellular electrical stimulation device’s assembling, a thin (thickness = 0.6 mm) PDMS film with circular hole (diameter = 18 mm) was laminated on clean glass substrate. Sequentially, the cellular electrical stimulation circuit was fixed on the sample with downward electrode by liquid PDMS. This structure will build a chamber between glass substrate and stimulation electrodes for cell culture and construction of electric field. Finally, a thick (thickness = 3 mm) PDMS film with circular hole (diameter = 18 mm) was laminated on cellular electrical stimulation circuit, which could ensure that stimulation circuit was immersed into cell culture fluid during in vitro experiments.

The Morphology of Dissociated HCECs Respond to Electric Fields

The suspension of human corneal epithelial cells (HCECs, ATCC, USA) was added to the self-made cell electrical stimulation (ES) device. The experimental group was stimulated by DC electric field of 100 mV / mm, and rested for 5 min every 15 min, for a total of 2 h. There was no electric stimulation applied for the control group. The morphology changes of HCECs were observed and captured by optical microscope. The cytoskeleton protein F-actin was labeled by DyLight™ 594 phalloidin (cell signaling technology #12877). Monoclonal Anti-α-Tubulin antibody (Sigma-Aldrich F2168) was used to stain tubulin protein in cells to clarify the morphology of cells and the direction of mitosis. E-Cadherin Mouse mAb (Cell signaling Technology #14472) was used to label epithelial cadherin to evaluate the degree of tight adhesion between HCECs. The above immunofluorescence staining progress were summarized as follows, fixed the cells with 4% paraformaldehyde for 15 min, gently rinsed them with PBS for three times, and used blocking buffer (1x PBS, 5% goat serum, 0.3% Triton™ X-100) blocked the cells for 60 min, added the diluted fluorescent labeled antibody and incubated overnight at 4 ℃. Gently rinse with PBS for three times, added diluted DyLight™ 594 phalloidin and incubate at room temperature for 15 min. After PBS cleaning, add Prolong® Gold antifade reagent with DAPI (# 8961), and then observed and recorded under fluorescence microscope.

HCECs Migration Assay

HCECs were cultured in the ES device until formed a monolayer after fusion, scratch wounds were made in the center by a cell scratch scraper (1 mm wide), and then observed and photographed under an optical microscope. The experimental group was stimulated by DC electric field of 100 mV / mm and rested for 5 min every 15 min. The control group did not do any treatment. Cultures were observed every 15 min until gap closure was occurred. Wound area was measured with ImageJ, and cell migration was determined as per cent of the wound area relative to the original wound area at the wound edge. The cytoskeleton protein F-actin was labeled by DyLight™ 594 phalloidin (cell signaling technology #12877). E-Cadherin Mouse mAb (Cell

signaling Technology #14472) was used to label epithelial cadherin to evaluate the degree of tight adhesion between HCECs. The steps of immunofluorescence staining are the same as before.

CCK-8 Assay

HCECs were seeded into 24-well culture plates, cultured at 37 °C with 5% CO_2, and the medium was replaced every day. The experimental group was stimulated by DC electric field of 100 mV / mm and rested for 5 min every 15 min. According to the total duration of electrical stimulation, it can be divided into: ES 1, ES 2 and ES 3, representing 1, 2 and 4 h respectively. The control group did not do any treatment. Cell activity was detected by a CCK-8 assay. CCK-8 solution was added to each well. After 4.5 h of incubation, the optical density, which representing cell viability was measured using a microplate spectrophotometer (Thermo Scientific, USA) at a test wavelength of 450 nm.

mRNA-Sequencing Test

The experimental group received electrical stimulation for 2 h (5 min interval following 15 min stimulation), while the control group received none treatment. Both groups of cells were washed gently with PBS three times, and then the cells were lysed using TRIzol. The extracted RNA was transferred to sterile, RNase-free EP tubes, quickly immersed in liquid nitrogen for storage, and then transported at low temperature to the BGI Group for mRNA detection and sequencing. We used the Dr. Tom software provided by the BGI Group to analyze the result data, and the differential gene detection was performed by the DEGSeq method. Genes with Q value < 0.05 and $|\log2|$ fold change ≥ 1 were screened as significant differential expressed genes (DEGs). Based on the analysis results, hierarchical clustering analysis was performed using R package heat map to achieve the concatenation of DEGs. Functional classification of DEGs was performed based on the results of Gene Ontology annotation as well as official classification. Meanwhile, enrichment analysis was performed using the Phyper function in R-software to calculate Q values.

Characterization of EBCL Promote Epithelial Healings

All animal experiments were conducted in strict accordance with the Association

for ARVO Statement Regarding to the Use of Animals in Ophthalmic and Vision Research. The content and design of all experiment were reviewed and permitted by Animal Experimental Ethics Committee of Zhongshan Ophthalmic Center at Sun Yat-sen University guidelines (No. o2022016). New Zealand rabbits (n=3) came from Huadong Xinhua Laboratory Animal Farm (Guangzhou, China) and raised in the standard environment for 7 days before experiment. The corneal wound was established in both eyes under anesthesia, the procedure was briefly described as follows: corneal wounds were produced by removing a circular region (3.5 mm diameter) of the central corneal epithelium with a cornea trephine. The dimension and depth of defect was kept consistent in both eyes and monitored by sodium fluorescein solution (0.1% in physiological saline solution). The corneal wounds were observed and photographed under diffuse illumination and cobalt-blue light by slit lamp microscope system. ON each experimental day, the right eye was treated with EBCL for electrical stimulation for 60 min per day (with1 min interval after every 15 min of stimulation) (950 kHz, high-level 185mv, low-level 0 mV, duty cycle 80%). The left eye was treated with general bandage contact lenses as the control. After treatments, the rabbits were returned to cage for regular accommodation. The corneal repair of both eyes was observed and recorded every day until one group exhibited wound healed. Healing was quantified as per cent of the wound area remaining at the end of the assay relative to the original wound area by Image J. After the final tracing, rabbits were euthanized and the entire cornea was removed. After fixed with 4% paraformaldehyde, the corneas paraffin section of both groups was made to observe the hematoxylin and eosin (HE) staining. Corneal epithelial cells were identified by immunofluorescence staining Anti-Cytokeratin 5 (CK5) antibody (Abcam, USA), cells tight adhesion was labeled by Anti-ZO1 tight junction antibody (Abcam, USA). Fibrillar collagen types were examined by Sirius red staining.

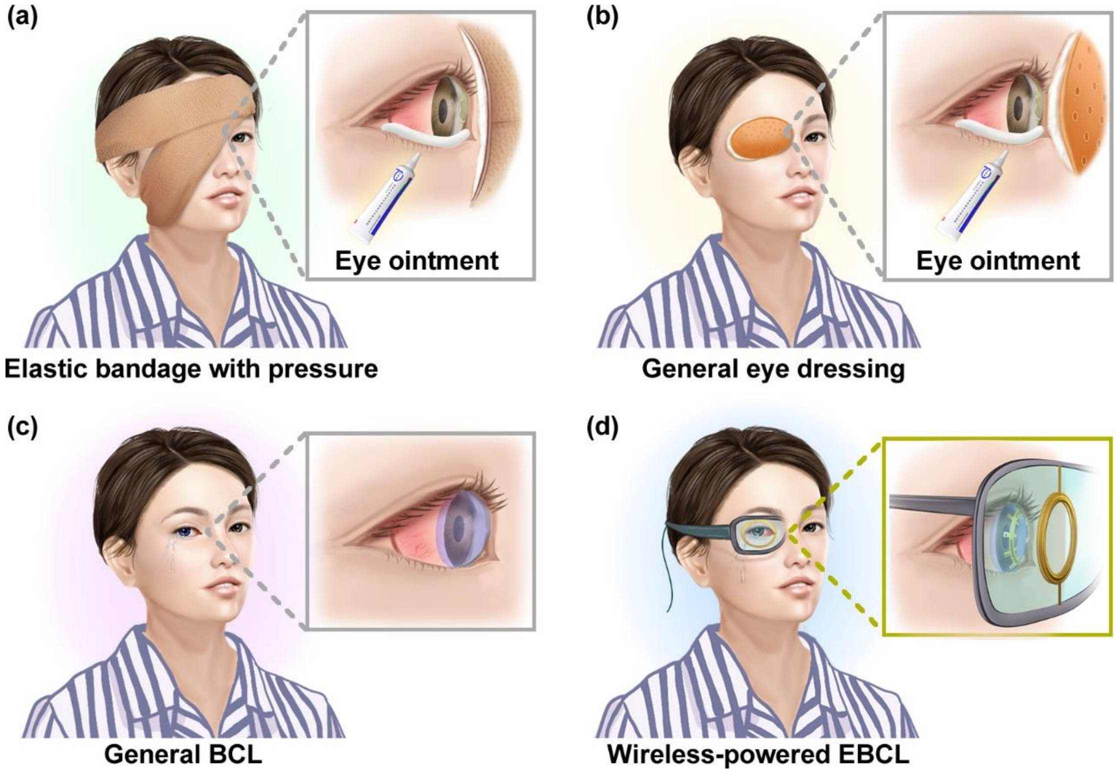

Figure S1. Corneal epithelial cell defects are often occurred after eye surgery or dry eye. Ocular compression bandaging after ointment administration is the main clinical treatment to promote corneal epithelial repair currently. However, in patients with persistent corneal epithelial defects, continuous dressing of ocular pressure bandaging is not conducive to wound observation and administration of eye drops. Bandage corneal lens (BCL) is an effective alternative to ocular compression bandages, but it is unable to participate in controlling cell behaviors. Our wireless-powered EBCL contained the following key advantages: 1) the bandage contact lens platform possessed features of softness, re-usable, and non-invasiveness, and wireless operations, which are compatible with the clinical applications and the daily life of patients; 2) Rational circuit designs and compact structural layout enable precise integration and on-demand electrical stimulation modulus in limited area of bandage contact lens without vision blockage; 3) The bandage system with electrical interface is desirable for programming and on-demand stimulation; 4) the fabrication process of the EBCL is compatible with existing circuit board manufacturing process, suggesting the potential for large-scale and cost-effective manufacturing.

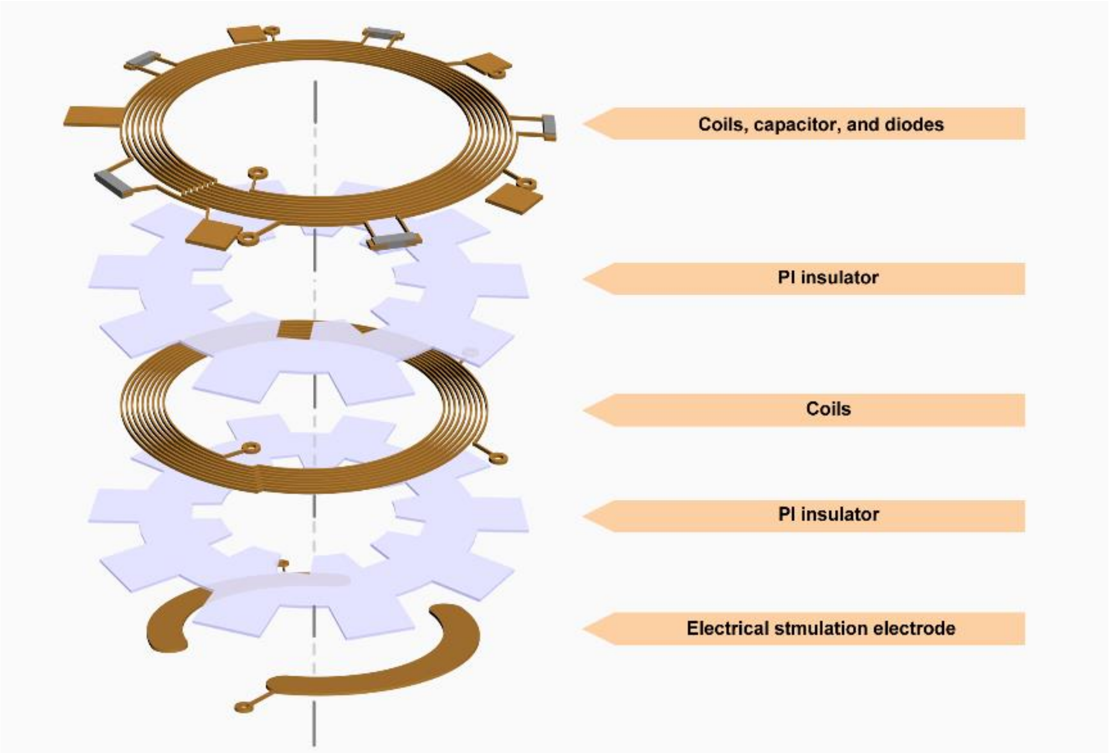

Figure S2. Structure of wirelessly electrical stimulation circuit.

Wirelessly electrical stimulation circuit adopted a multilayer structure that enabled an integration of wireless power transfer and rectify function on compact space. In this circuit, coils, capacitor, and diodes were deployed on top and middle layer of the circuit. And electrical stimulation electrodes were set on bottom of the circuit. Two layers of PI film were adopted as insulate layer between different conductive layer. Moreover, conductive through-holes were constructed to achieve electrical connection of three conductive layer.

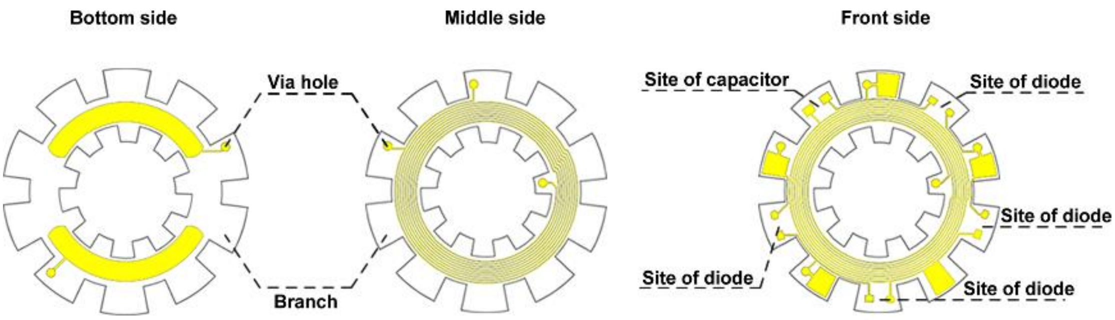

Figure S3. Accessorial design of the wirelessly electrical stimulation circuit. Conductive via hole constructed by laser and metal deposition have access to achieve electrical connections between different conductive layer. Moreover, multilayer ceramic chip capacitor and Schottky barrier diode chips were integrated on branch of flexible circuit by low temperature lead-free solder.

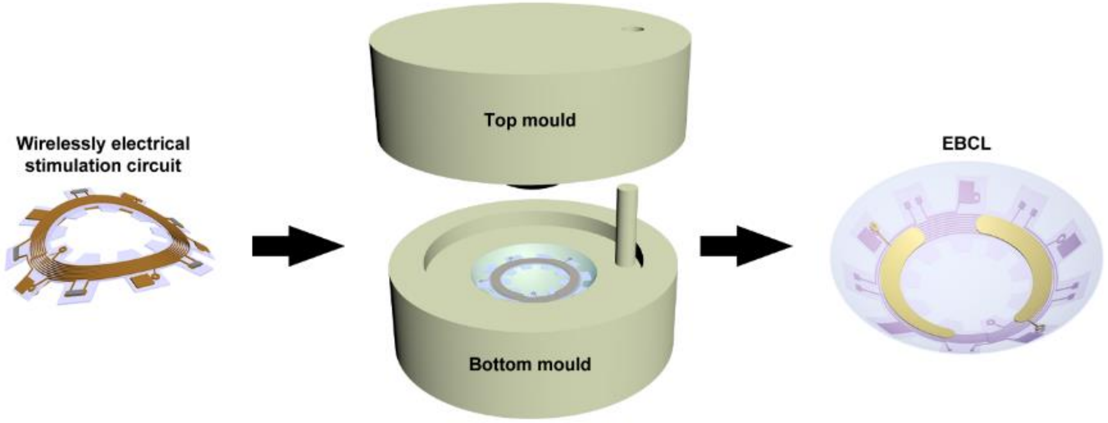

Figure S4. Integration process of wirelessly electrical stimulation circuit and contact lens.

Wirelessly electrical stimulation circuit was placed in bottom mould. After that, transparent liquids (Polydimethylsiloxane (PDMS, Sylgard 184, Dow Corning) and curing agent mixed according to the ration of 10:1) was mixed sufficiently by stirring and degassed by high-speed centrifugation. Then the transparent liquids were immitted into bottom mold. Sequentially, and the bottom mold contained wirelessly electrical stimulation circuit and liquid PDMS was placed in vacuum with pressure of 10 Pa for 30 min to remove residual bubbles. After vacuum treatment (10 Pa, 30 min), the mold was assembled and baked in oven with temperature of 80 ℃ for 1.5 h. Finally, the top and bottom mold were separated. And contact lens integrated with circuit was detached from mold carefully.

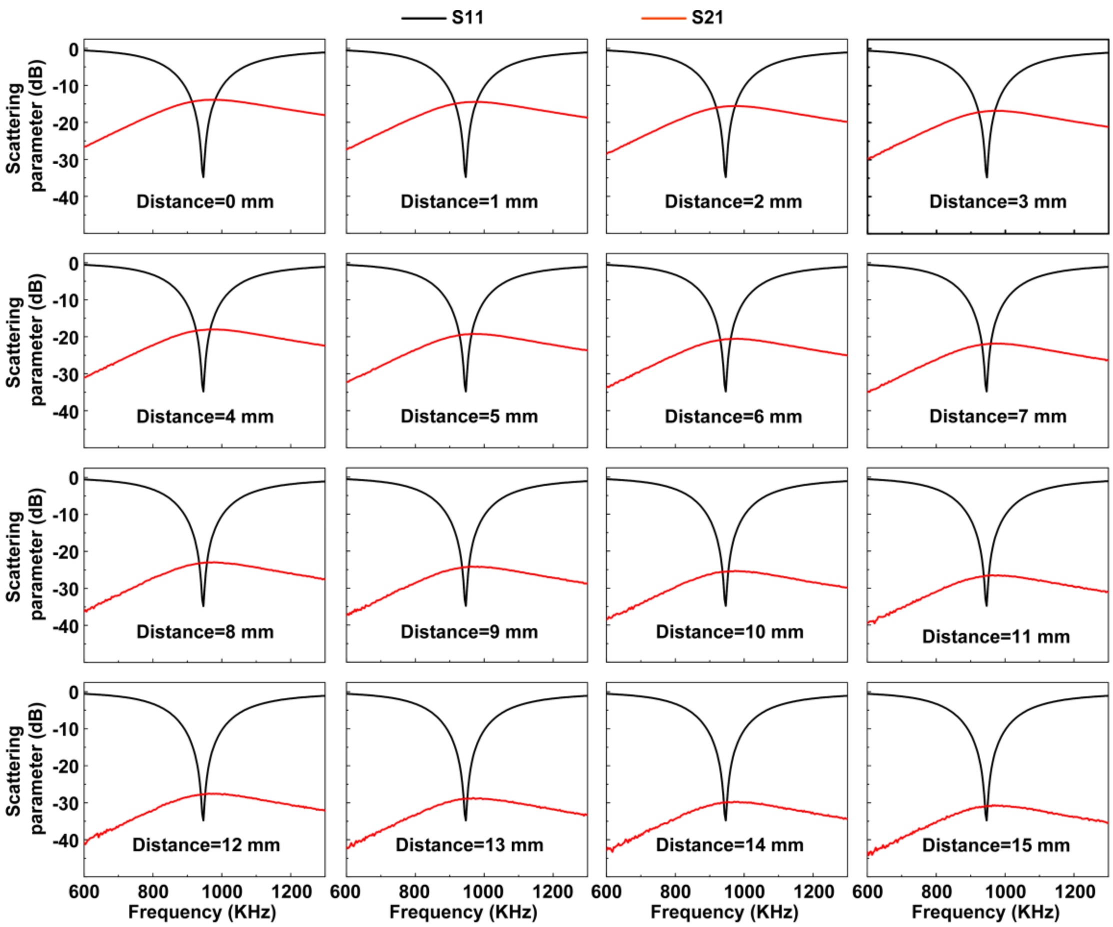

Figure S5. The scattering parameters of the wireless system.

The scattering parameters of the wireless system were recorded by vector network analyzer (Keysight E5063A) under different radiation distance from 0 mm to 15 mm with the step of 1 mm.

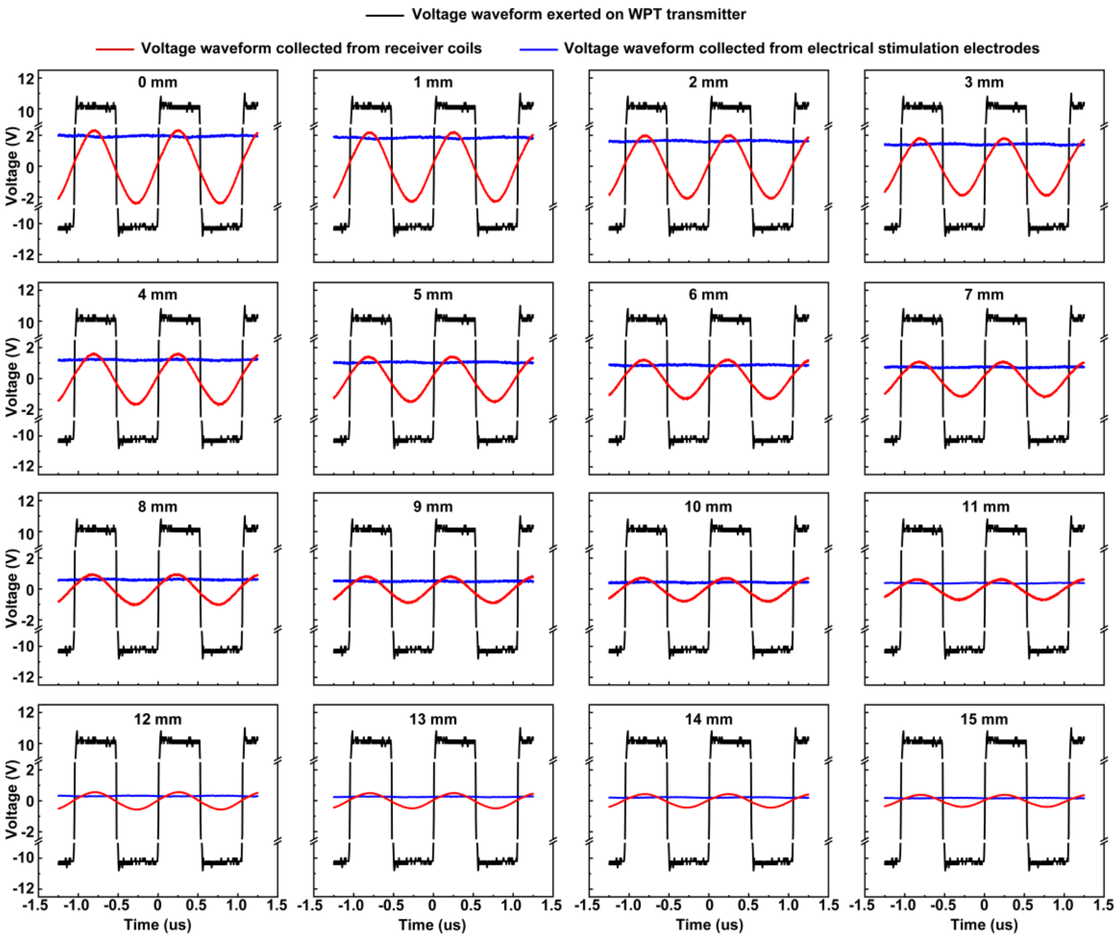

Figure S6. The influence of wireless distance for the voltage on coils and stimulation electrodes of EBCL.

Square voltage wave (black) with 20 Vpp was produced from waveform generator to WPT transmitter. Correspondingly, the voltage on the coils (red) and electrical stimulation electrodes (blue) of WPT receive circuit were recorded. Results demonstrated that the voltage collected on electrical stimulation electrodes of WPT receive circuit shown direct voltage features that was highly different from the sine shaped wave recorded from receiver coils.

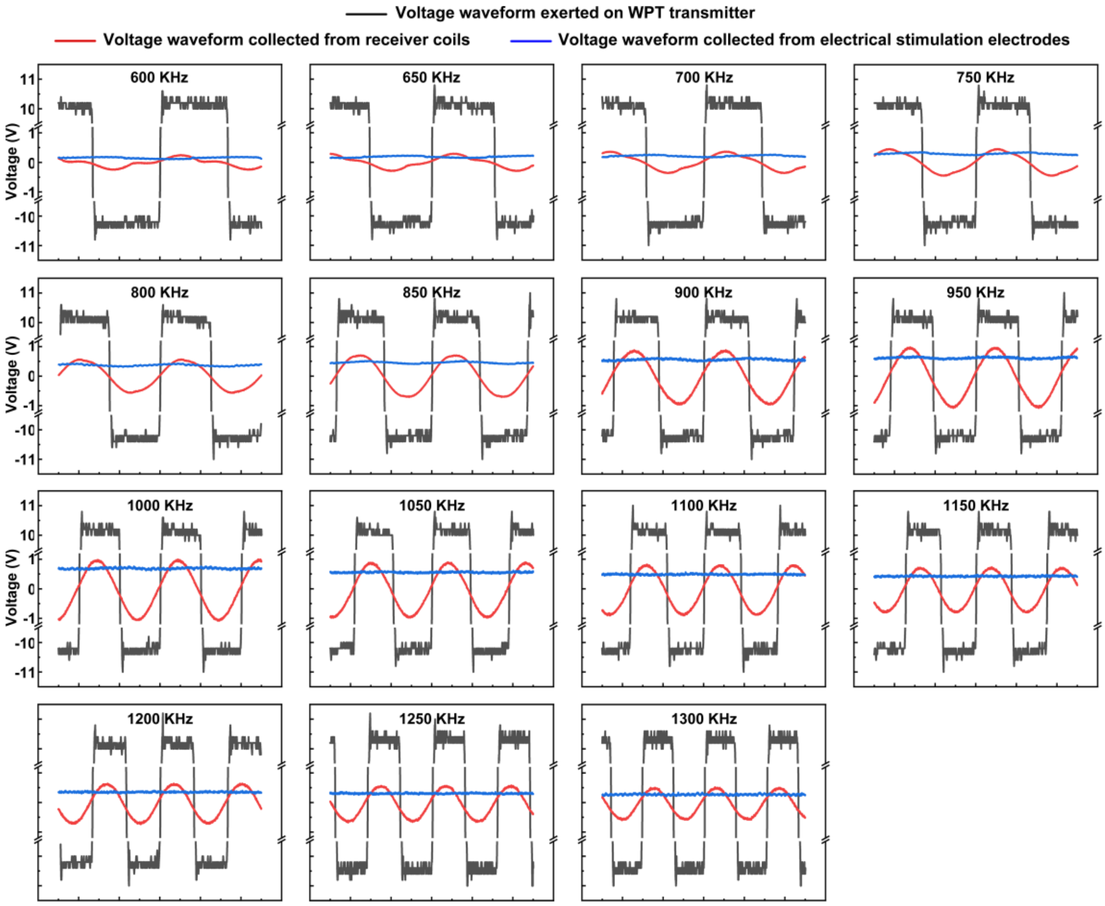

Figure S7. The influence of frequency for the voltage on coils and stimulation electrodes of EBCL.

Square voltage wave (black) with 20 Vpp was produced from waveform generator to WPT transmitter. Correspondingly, the voltage on the coils (red) and electrical stimulation electrodes (blue) of WPT receive circuit were recorded. Results demonstrated that the voltage collected on electrical stimulation electrodes of WPT receive circuit shown direct voltage features that was highly different from the sine shaped wave recorded from receiver coils.

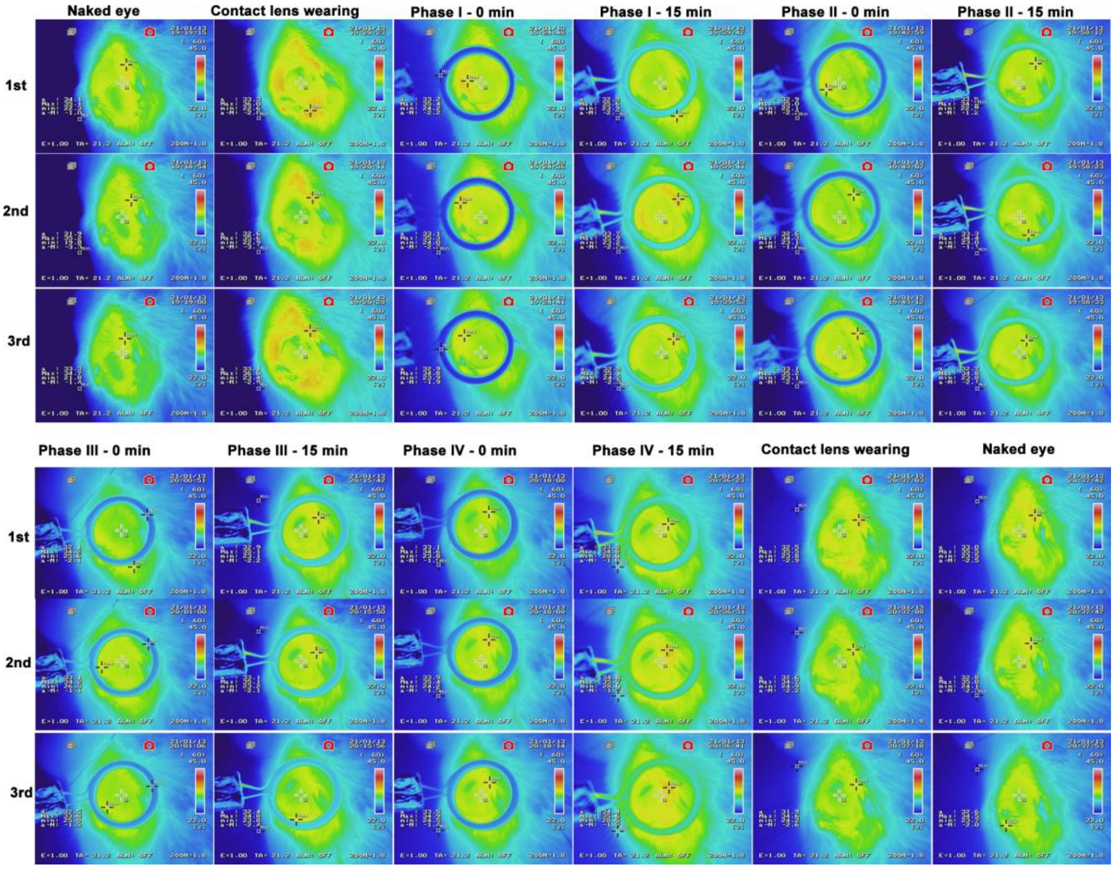

Figure S8. Detailedly infrared images of rabbit eye and bandage contact lens during animal experimentation.

EBCL was worn on anesthetized New Zealand white rabbit's eye. And wireless power transmitter connected to waveform generator was posited above EBCL with the distance of 6 mm. Square voltage with 20 Vpp at 950 kHz produced from waveform generator was exerted on wireless power transmitter. Infrared camera (T650sc, FLIR Systems, Wilsonille, OR, USA) was exploited to monitor thermal changes of ocular surface tissue, EBCL, and wireless power transmitter during the experimental process.

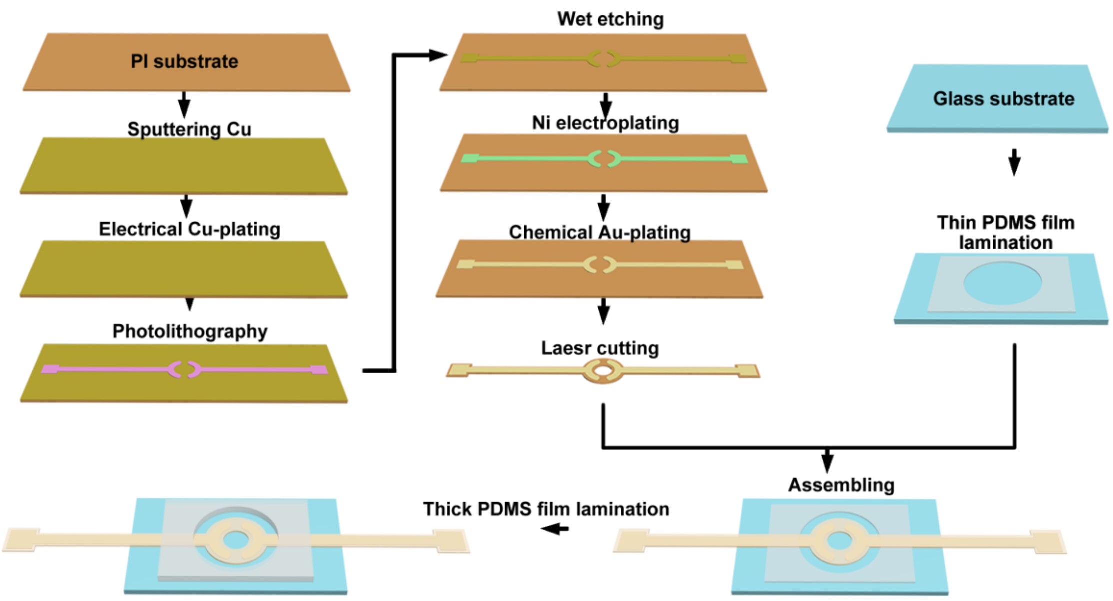

Figure S9. Fabricaiton of flexible circuit for assembling of electrical stimulation chamber.

As regards the fabrication process of flexible circuit for assembling of electrical stimulation chamber, Cu film with thickness of 100 nm was deposited on a clean polyimide (PI) substrate by magnetron sputtering. In order to acquire acceptable thickness, electrical plating process was adopted to elevate the thickness of Cu film. Then the Cu film was patterned via photolithography, and etched by $FeCl_3$ solution. After that, Cu film was covered with Ni/Au to enhance the biocompatibility of the circuit. Afterwards, PI substrate was shaped by laser to form cellular electrical stimulation circuit. Moreover, a thin (thickness = 0.6 mm) PDMS film with circular hole (diameter = 18 mm) was laminated on clean glass substrate. Sequentially, the cellular electrical stimulation circuit was fixed on the sample with downward electrode by liquid PDMS. This structure will build a chamber between glass substrate and stimulation electrodes for cell culture and construction of electric field. Finally, a thick (thickness = 3 mm) PDMS film with circular hole (diameter = 18 mm) was laminated on cellular electrical stimulation circuit, which could ensure that stimulation circuit was immersed into cell culture fluid during in vitro experiments.

Table S1. Detailed parameters of wirelessly electrical stimulation circuit.

Item	Parameter
Number of layers	3
Number of coil turns	16
Width of the inductance electrode (μm)	60
Capacitor matched with inductance coil (nF)	2.2
The inner diameter of circuit d_1 (mm)	5.6
The inner diameter of inductance coil d_2 (mm)	7.94
The outer diameter of inductance coil d_3 (mm)	10.61
The length of branch d_4 (mm)	1.5
The width of slit between branch d_5 (mm)	1.0
The central angle of electrical stimulation electrode	120°

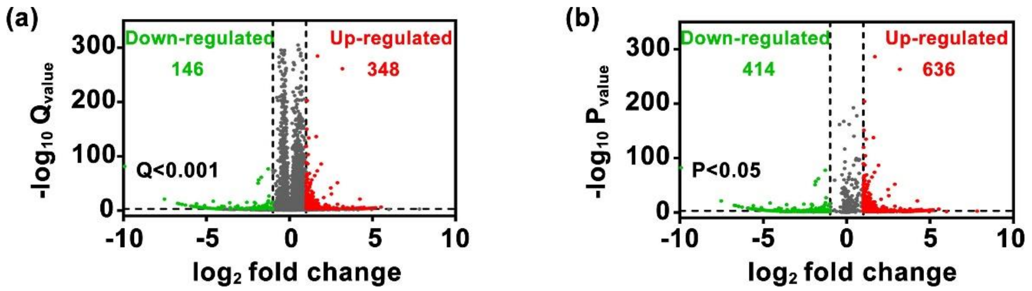

Figure S11. (a) Volcano plots displayed 348 genes significantly upregulated and 146 genes significantly downregulated whose Q value is <0.001 and | log 2 | fold change ≥ 1 compared the ES group with the control group. (b) Volcano plots displayed 636 genes significantly upregulated and 414 genes significantly downregulated whose P value is <0.05 and | log 2 | fold change ≥ 1 compared the ES group with the control group.

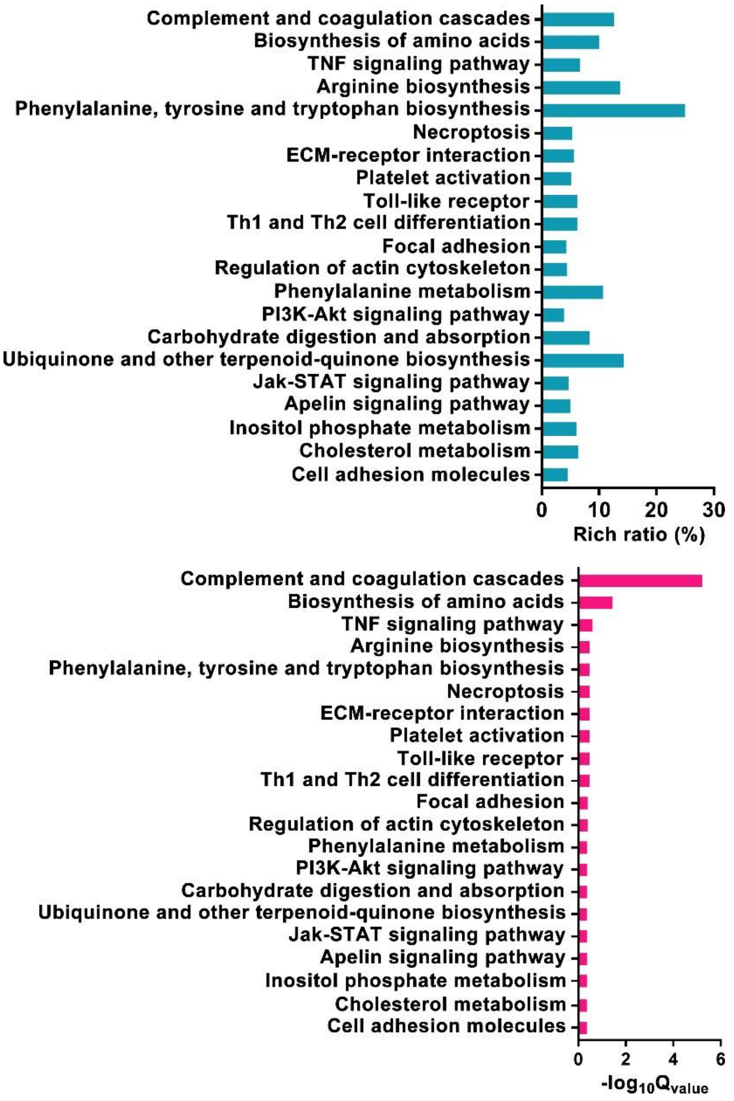

Figure S12. Rich ratio (%) and Q_{value} of genes in the enriched KEGG which are highly related in complement and coagulation cascades, biosynthesis of amino acids, TNF signaling pathway, arginine biosynthesis, Phenylalanine, tyrosine and tryptophan biosynthesis, necroptosis, ECM-receptor interaction, Toll-like receptor, Th1 and Th2 cell differentiation, focal adhesion, regulation of actin cytoskeleton, phenylalanine metabolism, PI3K-Akt signaling pathway, carbohydrate digestion and absorption, ubiquinone and other terpenoid-quinone biosynthesis, Jak-STAT signaling pathway, apelin signaling pathway , inositol phosphate metabolism, cholesterol metabolism , cell adhesion molecules pathways, whose enrichment rates exceed 3% with P_{value} <0.05.
